# Cigarette smoke enhances human rhinovirus-induced CXCL8 production via HuR-mediated mRNA stabilization in human airway epithelial cells

**DOI:** 10.1186/1465-9921-14-88

**Published:** 2013-08-30

**Authors:** Magdalena H Hudy, David Proud

**Affiliations:** 1 University of Calgary Faculty of Medicine, HRIC 4C50-54 3280 Hospital Drive N.W., Calgary, AB T2N 4Z6, Canada; 2 Department of Physiology & Pharmacology, HRIC 4AC60, University of Calgary Faculty of Medicine, 3280 Hospital Drive N.W., Calgary, AB T2N 4Z6, Canada; 3 Airways Inflammation Research Group, Snyder Institute for Chronic Diseases, University of Calgary Faculty of Medicine, Calgary, AB Canada

**Keywords:** Airway epithelium, Rhinovirus, Cigarette smoke, CXCL8, HuR, Post-transcriptional gene regulation, COPD, Asthma

## Abstract

**Background:**

Human rhinovirus (HRV) triggers exacerbations of asthma and chronic obstructive pulmonary disease (COPD). Cigarette smoking is the leading risk factor for the development of COPD and 25% of asthmatics smoke. Smoking asthmatics have worse symptoms and more frequent hospitalizations compared to non-smoking asthmatics. The degree of neutrophil recruitment to the airways correlates with disease severity in COPD and during viral exacerbations of asthma. We have previously shown that HRV and cigarette smoke, in the form of cigarette smoke extract (CSE), each induce expression of the neutrophil chemoattractant and activator, CXCL8, in human airway epithelial cells. Additionally, we demonstrated that the combination of HRV and CSE induces expression of levels of CXCL8 that are at least additive relative to induction by each stimulus alone, and that enhancement of CXCL8 expression by HRV+CSE is regulated, at least in part, via mRNA stabilization. Here we further investigate the mechanisms by which HRV+CSE enhances CXCL8 expression.

**Methods:**

Primary human bronchial epithelial cells were cultured and treated with CSE alone, HRV alone or the combination of the two stimuli. Stabilizing/destabilizing proteins adenine/uridine-rich factor-1 (AUF-1), KH-type splicing regulatory protein (KHSRP) and human antigen R (HuR) were measured in cell lysates to determine expression levels following treatment. siRNA knockdown of each protein was used to assess their contribution to the induction of CXCL8 expression following treatment of cells with HRV and CSE.

**Results:**

We show that total expression of stabilizing/de-stabilizing proteins linked to CXCL8 regulation, including AUF-1, KHSRP and HuR, are not altered by CSE, HRV or the combination of the two stimuli. Importantly, however, siRNA-mediated knock-down of HuR, but not AUF-1 or KHSRP, abolishes the enhancement of CXCL8 by HRV+CSE. Data were analyzed using one-way ANOVA with student Newman-Keuls post hoc analysis and values of p≤ 0.05 were considered significant.

**Conclusions:**

Induction of CXCL8 by the combination of HRV and CSE is regulated by mRNA stabilization involving HuR. Thus, targeting the HuR pathway may be an effective method of dampening CXCL8 production during HRV-induced exacerbations of lower airway disease, particularly in COPD patients and asthmatic patients who smoke.

## Background

More than half of all exacerbations of asthma and COPD are associated with viral infections, with HRV being the dominant viral pathogen detected [1]. *In vivo*, the airway epithelium is the primary site of HRV infection but infection does not induce any obvious cytopathic effects in epithelial cells. Rather, it is now generally thought that HRV alters epithelial cell biology in a manner which results in a virally-induced enhanced inflammatory state [2,3]. In support of this, it is well established that HRV infection of human bronchial epithelial cells, both *in vivo* and *in vitro*, triggers the release of a variety of pro-inflammatory and host-defence genes [3,4].

Cigarette smokers experience more frequent upper respiratory infections that both last longer and are more severe, when compared to non-smokers [5-8]. Moreover, cigarette smoke generally impairs innate immune responses [9-13], including during viral infections with influenza [14-18] or respiratory syncytial virus [19,20]. Approximately a quarter of patients with asthma smoke, and these individuals have worse respiratory symptoms, require more hospitalizations and are less responsive to anti-inflammatory treatments than asthmatic patients who do not smoke [21,22]. Cigarette smoking is the dominant risk factor associated with the development and progression of COPD. Taken together, these observations suggest that HRV infection in COPD patients, and in asthmatic patients who smoke, would lead to substantially worse clinical outcomes and exacerbations compared to their non-smoking counterparts.

There is now growing evidence that CSE modulates HRV induced expression of many inflammatory, antiviral and host defence genes in airway epithelial cells [23-26]. Although in many cases, CSE down-regulates HRV-induced epithelial gene expression, this is not the case for the potent neutrophil chemoattractant, CXCL8 [23,24]. Rather, we have shown that HRV and CSE each alone induce production of CXCL8 and the combined stimulus induces at least additive production of CXCL8 from human bronchial epithelial cells [24]. This may have important consequences as levels of CXCL8 have been shown to correlate with symptom severity during HRV infections [27]. Moreover, not only do neutrophil numbers in sputum correlate with disease severity in patients with COPD [28], but neutrophil numbers and neutrophil degranulation correlate with disease severity during viral exacerbations of asthma and COPD [29,30]. In addition, increased sputum levels of CXCL8 are associated with neutrophilic inflammation in asthmatics who smoke [22].

We have shown that the enhancement of HRV-induced epithelial CXCL8 production by CSE is due, at least in part to mRNA stabilization [24]. It is well established that stability of mRNA encoding CXCL8 can be regulated via effects at adenine/uridine-rich elements (AREs) present in the 3’ UTR of CXCL8 mRNA [31,32]. Four elements composed of AUUUA are present in the proximal portion of the CXCL8 3’ UTR, two of which are overlapping [33-35]. CXCL8 mRNA stability has been shown to be regulated via the p38 MAPK pathway [35-37] and we have shown that the combination of HRV+CSE induces activation of the p38 MAPK pathway and that inhibition of this pathway attenuates the additive enhancement of CXCL8 by HRV+CSE compared to either treatment alone [24].

Several mRNA stabilizing/destabilizing proteins have been reported to be involved in regulating the degradation rate of CXCL8 mRNA, depending on the cell type and stimuli studied, with no overall consensus of which is the dominant factor involved in mRNA stability of this gene. These include AUF-1 [33], KHSRP [38,39], HuR [33-35,38] and tristetraprolin (TTP) [23,34]. HuR is involved in stabilizing mRNA, KHSRP and TTP both destabilize mRNA, while AUF-1, which has four isoforms and has been shown to both stabilize and destabilize mRNA transcripts [40]. To date none of these proteins have specifically been linked to CSE-induced stabilization of CXCL8. Moreover, the mechanisms responsible for regulating mRNA stabilization in epithelial cells exposed to HRV and CSE are unknown. We hypothesized that the enhancement of HRV-induced CXCL8 by CSE is regulated via the actions of one or more of these proteins, and the current study was performed to test this hypothesis.

## Methods

### Materials

The following reagents were purchased from the indicated suppliers: bronchial epithelial cell basal medium and additives to create serum-free bronchial epithelial cell growth medium (BEGM) (Lonza, Walkersville, MD); WI-38 cells and HRV type 16 (American Type Culture Collection, Manassas, VA); 3R4F research grade cigarettes (College of Agriculture Reference Cigarette Program, University of Kentucky) firefly luciferase reporter plasmid pGL4.10[*luc*2] and passive lysis buffer (Promega, Madison, WI); firefly luciferase assay kit (Biotium Inc., Hayward, CA); TransIT-LT1 transfection reagent (Mirus, Madison, WI); anti-AUF-1 antibody (#07-260, Upstate curtsey of EMD Millipore, Billerica, MA); anti-KHSRP antibody (#Ab83291, Abcam, Toronto, ON, Canada); anti-HuR antibody (#A-21277, Molecular Probes courtesy of Life Technologies, Burlington, ON, Canada); glyceraldehyde-3-phosphate-dehydrogenase (GAPDH) antibody (AbD Serotec, Raleigh, NC); horseradish peroxidase (HRP)-conjugated anti-mouse antibody (Jackson ImmunoResearch Laboratories, West Grove, PA); HRP-conjugated anti-rabbit antibody (GE Healthcare Biosciences, Piscataway, NJ); enhanced chemiluminescent (ECL) substrate reagent (GE Healthcare Biosciences, Piscataway, NJ); Lowry DC protein assay (Biorad Laboratories, Mississauga, ON); recombinant CXCL8 protein (R & D Systems, Minneapolis, MN); siRNA targeting AUF-1 (S100300454 and S102653665), KHSRP (S100300587 and S100054691) and HuR (S100300139 and S103246551) (Qiagen, Toronto, ON, Canada); medium GC negative control non-targeting siRNA, OptiMEM reduced serum media and Lipofectamine RNAiMAX transfection reagent (Invitrogen, Burlington, ON, Canada). All other chemicals were purchased from Sigma-Aldrich (Oakville, ON, Canada).

### Epithelial cell culture

Primary human bronchial epithelial (HBE) cells were derived using previously described methods [41]. Briefly, cells were obtained via protease digestion of dissected airways from normal non-transplanted human lungs obtained from a tissue retrieval service (International Institute for the Advancement of Medicine (IIAM), Edison, NJ). Ethical approval to receive and utilize lung tissues was obtained from both the Conjoint Health Research Ethics Board of the University of Calgary (Calgary, AB) and from the Internal Ethics Board of IIAM (Edison, NJ). No personal identifying information was provided for any of the donors. For these studies, cells were derived from 6 individual lung donors. Five donors were male and ages ranged from 20 to 62 years. All subjects died of head trauma or from cerebrovascular causes. HBE cells were cultured in BEGM at 37°C in 5% CO_2_. Prior to experimental treatments, cells were cultured overnight in BEGM from which hydrocortisone was removed (BEGM no HC), and then that medium was used for subsequent experiments.

### Preparation of purified HRV and CSE

HRV-16 stocks were propagated in WI-38 fibroblast cells and purified by density centrifugation on a sucrose cushion as previously described [42]. CSE was prepared according to previously described methods [24]. Briefly, CSE was generated by bubbling one 3R4F research grade cigarette into 4 mL of BEGM without hydrocortisone at a rate of 5 min per cigarette using a syringe apparatus. The resulting solution was filtered through a 0.22 μm filter to remove bacteria and/or large particles and subsequently adjusted with medium to an absorbance reading of 0.15 at 320 nm. This was arbitrarily defined as 100% CSE. Based on previous viability studies, CSE concentrations at or below an absorbance reading of 0.075 did not affect cell viability of HBE cells as assessed by the MTT viability assay [24]. Therefore, we used 50% CSE (absorbance of 0.075 at 320 nm) for all exposures in the current study. This final dilution of CSE applied to cells represented a 1:20 final dilution of the original 4 ml extract, and represents approximately 1/80^th^ of the soluble components of a single cigarette per well of a 6-well plate.

### HRV infection and CSE treatment

HBE cells were infected with 10^5.5^ tissue culture-infective dose U/ml (multiplicity of infection ~1.0) of purified HRV. Cells were treated with CSE alone, HRV alone or with CSE and HRV together, and were then incubated at 34°C in 5% CO_2_.

### Lactate dehydrogenase (LDH) assay

The viability of cells was also assessed using the Cyto96™ LDH assay according to the manufacturer’s instructions. Data were expressed as percent cytotoxicity of HBE cells following treatment with CSE alone, HRV alone or HRV+CSE compared to HBE cells treated with medium alone.

### siRNA transfections

Sub-confluent HBE cells were used for transient siRNA transfections. Individual siRNAs were diluted in serum-free OptiMEM media and transfected using Lipofectamine RNAiMAX reagent according to the manufacturer’s specifications. Transfection reagent only, and non-targeting siRNA controls were used for each treatment. HBE cells were treated with 1:4 siRNA-transfection lipid mixture and 3:4 BEGM without antibiotics (no PSF, gentamicin or amphotericin) with a final siRNA concentration of either 10nM (HuR and KHSRP) or 30nM (AUF-1) siRNA and incubated for 24 h at 37°C and 5% CO_2._ The supernatant was then aspirated and cells were recovered in BEGM no HC for 24 h at 37°C and 5% CO_2_. Following recovery, cells were subject to the desired treatment for an additional 24 h at 34°C and 5% CO_2_.

### Western blotting and ELISAs

Following appropriate treatments, supernatant was removed and cells were lysed with ice-cold lysis buffer (1% Triton X-100 in 1X MES buffered saline pH 7.4, anti-protease tablets, 50 nM sodium orthovanadate, 0.4M sodium pyrophosphate, 1M sodium fluoride and 100 mM phenylmethanesulfonylfluoride). Cells were scraped off the plate, frozen overnight to enhance cell lysis, and centrifuged. Total protein concentration in cell lysates was quantified using the Lowry DC protein assay (Bio-Rad, Mississauga, ON, Canada). Equivalent amounts of each sample (10 μg total protein) were separated by SDS-PAGE and proteins were then transferred to a 0.45 μm nitrocellulose membrane. Membranes were blocked with 5% skim milk for 1 h and incubated with an appropriate dilution of specific primary antibody (1:1000 for anti-AUF-1, 1 μg/mL for anti-KHSRP and anti-HuR) at 4°C overnight. Membranes were then washed, incubated with either HRP-conjugated anti-mouse (1:2000 following anti-HuR) or anti-rabbit (1:1000 following anti-AUF-1 and 1:10,000 following anti-KHSRP) secondary antibody for 1 h, washed again and developed using ECL substrate reagent. Re-probing stripped membranes with an antibody to the housekeeping gene GAPDH confirmed equal loading of protein. Densitometric analysis was performed using ImageJ software (version 1.41, National Institute of Health, Bethesda, MD, USA). Percent expression of the protein of interest was assessed by comparison to the appropriate control and normalized for minor protein loading variation to GAPDH levels.

Secreted CXCL8 was assessed in cell supernatants by ELISA assay using previously described methods [4]. The minimum level of detection for CXCL8 was 30pg/mL.

### Statistical analysis

Normal distribution of data was assessed using the Kolmogorov-Smirnov normality test. Data were analyzed using one-way ANOVA with student Newman-Keuls post hoc analysis. Values of p≤ 0.05 were considered significant.

## Results and discussion

### CSE alone, or in combination with HRV, does not affect HBE cell viability

**Figure 1:**
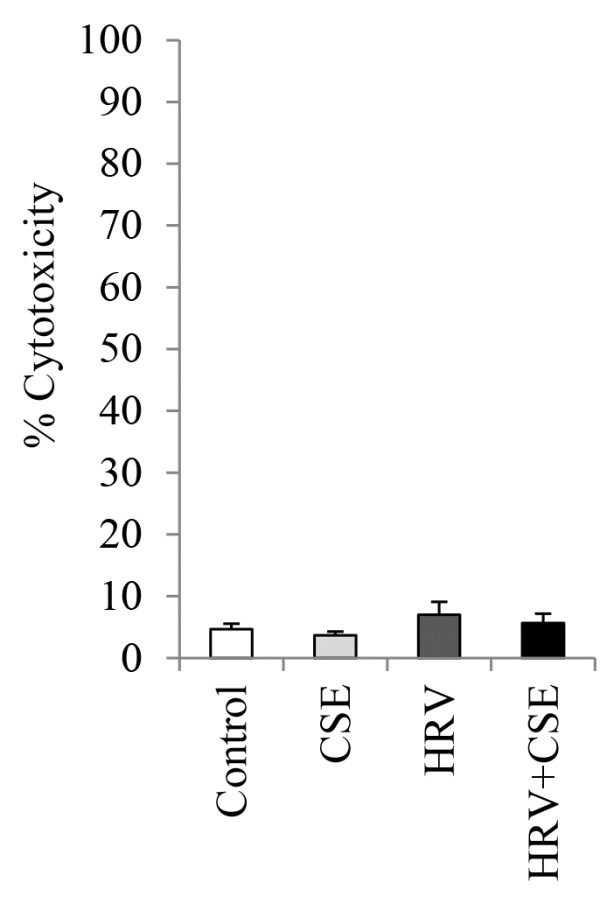
**CSE alone, HRV alone, or HRV+CSE do not affect HBE cell viability.** HBE cells were treated with medium (control), CSE, HRV or the combination for 24h prior to viability assessment using the LDH assay. Data are presented as mean ± SEM (n=3).

We have previously shown that the concentration of CSE utilized for these studies, either alone or in combination with HRV, does not affect HBE cell viability as assessed by the MTT assay [24]. Here, we confirmed that cell viability was also unaffected as assessed via the LDH assay in HBE cells treated with CSE alone, HRV alone or HRV+CSE (Figure 1).

### Expression levels of mRNA stabilizing/destabilizing proteins AUF-1, KHSRP and HuR are not altered by CSE, HRV or HRV+CSE at early time-points

**Figure 2:**
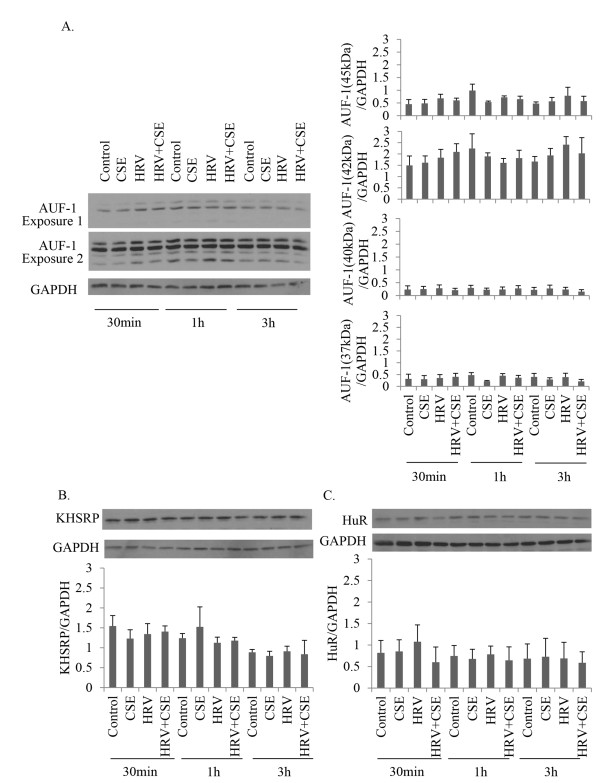
**CSE, HRV, or HRV+CSE do not alter AUF-1, KHSRP and HuR expression in HBE cells.** HBE cells were treated with medium (control), CSE, HRV or the combination for the times indicated. Total cell lysates were harvested and analyzed via immunoblotting. Membranes were probed with specific AUF-1 **(A)**, KHSRP **(B)** or HuR **(C)** antibodies, then were subsequently stripped and re-probed with an antibody to GAPDH to ensure equal protein loading. Data are representative of 3 separate experiments. Relative expression levels of each protein of interest from the 3 experiments were assessed by densitometry.

To investigate the roles of the mRNA stabilizing/de-stabilizing proteins that have been previously linked to CXCL8 mRNA stabilization, we first determined whether the expression of these proteins was altered in primary HBE cells stimulated with CSE alone, HRV alone or HRV+CSE. Using the actinomycin D chase assay, mRNA stabilization of CXCL8 by HRV+CSE was observed within 3 h post-treatment [24]. Therefore, we examined the expression of these stabilizing/destabilizing proteins at early time-points, as this would be the most relevant time-frame to affect the stabilization of CXCL8 mRNA. Although there was constitutive expression of AUF-1 (Figure 2A), KHSRP (Figure 2B) and HuR (Figure 2C) at 30 min, 1 h and 3 h, none of the treatments visually affected the expression level of these proteins in HBE cells at the time-points studied. Moreover, there was no significant difference in expression levels of these proteins as assessed by densitometry (Figure 2A-C). Examination of AUF-1 and HuR expression at later time-points, including 6 and 9 h post treatment with CSE, HRV or HRV+CSE resulted in similar observations, with no difference in expression levels of these proteins following treatment (data not shown). Using three different antibodies, we were unable to detect TTP protein in the HBE cells, either constitutively or following any of the treatments; therefore it is unlikely that the expression of TPP would be contributing to CXCL8 gene regulation by HRV+CSE. Two of these antibodies readily detected stimulus-induced TTP in the bronchial epithelial BEAS-2B cell line (data not shown). In contrast to our data in primary HBE cells derived from normal subjects, TTP has been shown to be expressed at very low levels in cystic fibrosis lung epithelial cells at rest. Upon stimulation it has been shown to regulate CXCL8 mRNA expression in these cells [43,44]. The discrepancy in these results could be attributed to the use of cystic fibrosis cells as opposed to epithelial cells derived from normal individuals, as it is possible that TTP expression is enhanced to detectable levels in disease states. Collectively, these data imply that, if AUF-1, KHSRP or HUR are involved the enhancement of HRV-induced CXCL8 by CSE in HBE cells, it is not via modulation of their expression levels.

### Enhancement of HRV-induced CXCL8 by CSE is regulated via HuR

**Figure 3:**
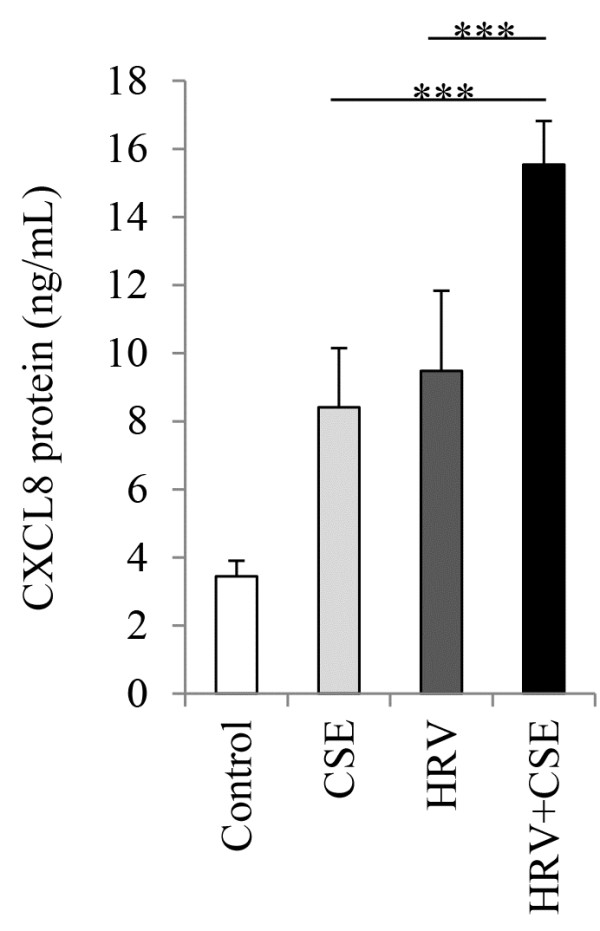
**CXCL8 protein is enhanced by HRV+CSE compared to CSE or HRV alone in HBE cells.** HBE cells from 6 HBE cell donors to be used for siRNA experiments were treated with medium control, CSE, HRV or HRV+CSE for 24 h and cell supernatants were assessed for CXCL8 protein release. Data are presented as mean ± SEM (n=6). Asterisks denote a significant difference between the specified treatments (*** p<0.001).

Although expression levels of AUF-1, KHSRP or HuR were not altered, it remained possible that treatment with CSE, HRV or HRV+CSE could affect the functions of these proteins within the cell. The activity of TTP, KHSRP and HuR all can be regulated by phosphorylation [45-47]. Unfortunately, there are no currently available commercial antibodies which selectively identify post-translational modifications of AUF-1, KHSRP or HuR. To further assess the role of these stabilizing/destabilizing proteins in enhancement of HRV-induced CXCL8 by CSE, we used an alternative approach utilizing siRNA to target the knock-down of each of AUF-1, KHSRP and HuR. As a reference to compare effects of siRNA knockdown on levels of CXCL8, the levels produced by the same cell preparations exposed to HRV alone, CSE alone or the combination of the two, are shown in Figure 3. This data also confirms our previously published results in HBE cells obtained from an additional 6 primary cell donors [24].

**Figure 4:**
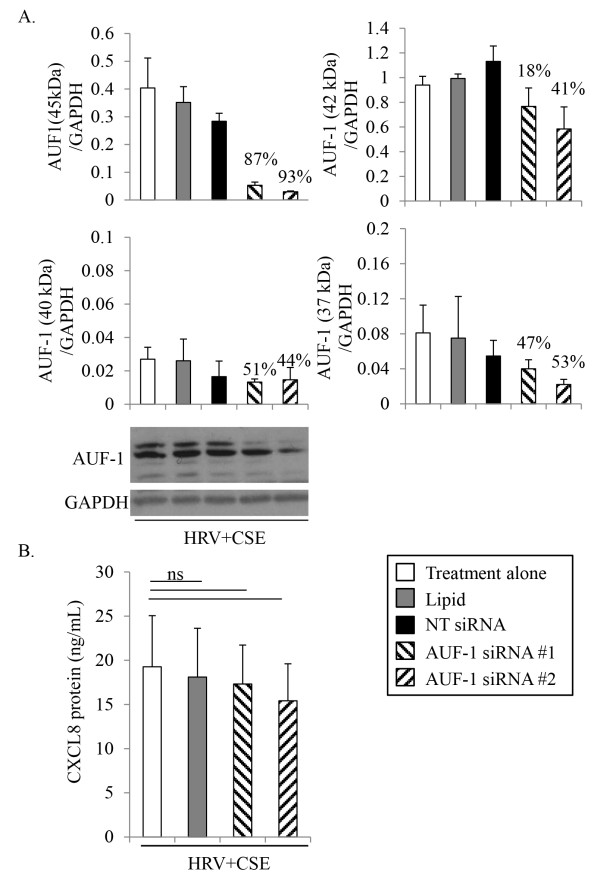
**Effects of AUF-1-targeting siRNA on CXCL8 protein expression in HBE cells.** HBE cells were treated for 24 h prior to harvesting cell supernatants for analysis with ELISA and whole cell lysates for analysis with immunoblotting. To determine the level of AUF-1 protein knock-down membranes were probed with a specific AUF-1 antibody recognizing 4 isoforms of AUF-1, then were subsequently stripped and re-probed with an antibody to GAPDH to ensure equal protein loading **(A**; representative of 3 separate experiments**)**. Densitometry analysis with % knock-down relative to HRV+CSE alone is also shown for each isoform of AUF-1 **(A**; n=3**)**. Supernatants were analyzed for CXCL8 protein **(B**; n=3**)**. Data are presented as mean ± SEM. Lipid denotes transfection reagent alone. NT denotes non-targeting control siRNA. ns = not significant.

**Figure 5:**
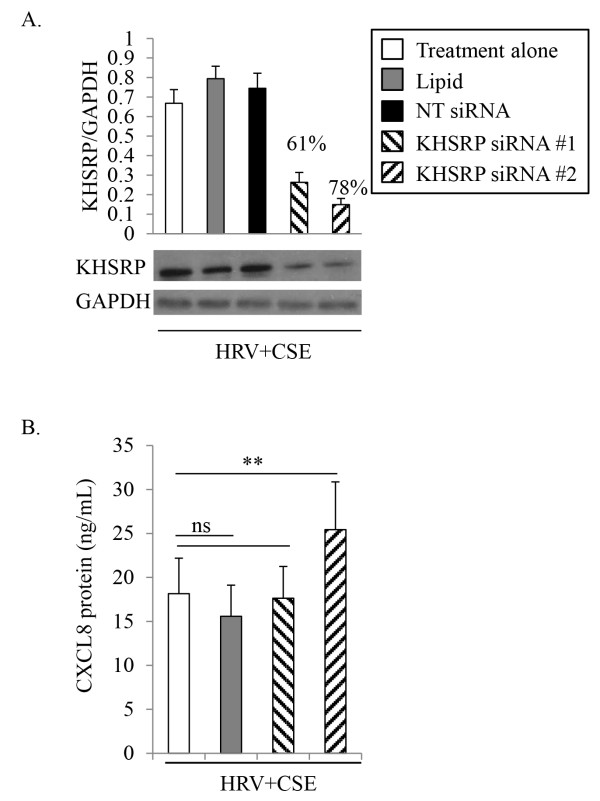
**Effects of KHSRP-targeting siRNA on CXCL8 protein expression in HBE cells.** HBE cells were treated for 24 h prior to harvesting cell supernatants for analysis with ELISA and whole cell lysates for analysis with immunoblotting. To determine the level of KHSRP protein knock-down membranes were probed with a specific KHSRP antibody, then were subsequently stripped and re-probed with an antibody to GAPDH to ensure equal protein loading **(A**; representative of 3 separate experiments**)**. Densitometry analysis with % knock-down relative to HRV+CSE alone is also shown **(A**; n=3**)**. Cell supernatants were analyzed for CXCL8 protein **(B**; n=5**)**. Data are presented as mean ± SEM. Lipid denotes transfection reagent alone. NT denotes non-targeting control siRNA. Asterisks denote a significant difference between the specified treatments (**p<0.01). ns = not significant.

**Figure 6:**
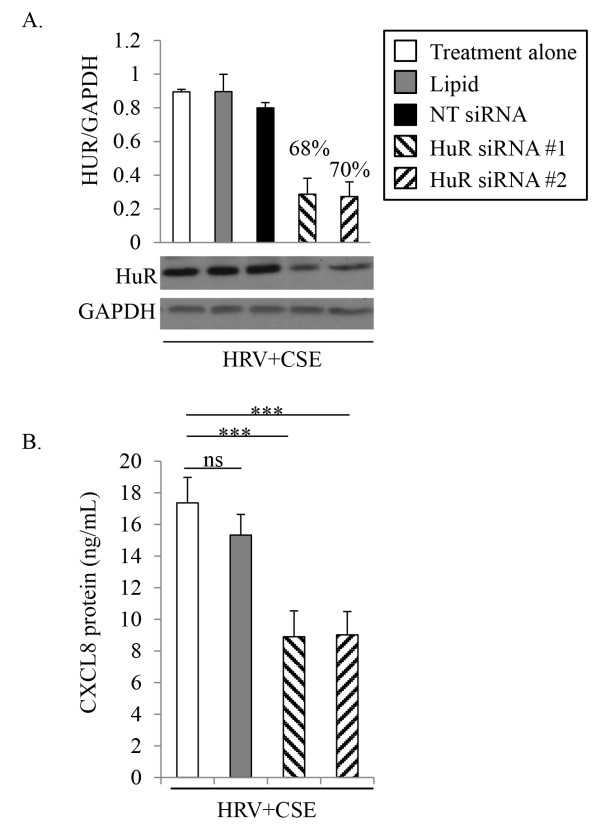
**Effects of HuR-targeting siRNA on CXCL8 protein expression in HBE cells.** HBE cells were treated for 24 h prior to harvesting cell supernatants for analysis with ELISA and whole cell lysates for analysis with immunoblotting. To determine the level of HuR protein knock-down membranes were probed with a specific HuR antibody, then were subsequently stripped and re-probed with an antibody to GAPDH to ensure equal protein loading **(A**; representative of 3 separate experiments**)**. Densitometry analysis with % knock-down relative to HRV+CSE alone is also shown **(A**; n=3**)**. Cell supernatants were analyzed for CXCL8 protein **(B**; n=6**)**. Data are presented as mean ± SEM. Lipid denotes transfection reagent alone. NT denotes non-targeting control siRNA. Asterisks denote a significant difference between the specified treatments (***p<0.001).

Primary HBE cells were transfected with each of two different siRNA duplexes targeting either AUF-1 (Figure 4), KHSRP (Figure 5) or HuR (Figure 6). Appropriate controls were performed in parallel; including treatment with medium alone, mock transfection with the transfection lipid alone, and transfection with the appropriate concentration of a non-targeting control siRNA, as not all siRNAs were effective at the same concentration. Cells were recovered for 24 h prior to an additional 24 h treatment with HRV+CSE. Initial studies focused on validation of respective protein knockdown. Although there was basal expression of AUF-1, KHSRP and HuR in HBE cells, we confirmed siRNA knock-down following our treatment of interest (HRV+CSE) in order to ensure that this treatment was not limiting the ability of the siRNAs to successfully produce knockdown. For all siRNA validation experiments (Figures 4A, 5A and 6A), similar levels of knockdown were observed in cells treated with medium alone, CSE alone or HRV alone (data not shown).

The AUF-1 gene is expressed as four alternatively spliced products with differing molecular weights, including 37, 40, 42 and 45 kDa isoforms. Neither the transfection reagent alone nor the control non-targeting siRNA (30 nM) had a significant effect on protein expression of any AUF-1 isoform (Figure 4A). Despite using AUF-1-targeting siRNA duplexes from two different suppliers targeting different regions of the molecules, we were unable to effectively knockdown all four AUF-1 isoforms. The reasons for this are unclear. Each of the two siRNAs targeting AUF-1 was only able to substantially knockdown the expression of the 45 kDa isoform of AUF-1. Densitometry calculations showed that the knockdown of the 45 kDa isoform was around 90% with each of the two siRNAs while knockdown of the other three isoforms was not as efficient (Figure 4A). Next, CXCL8 protein levels were measured from supernatants collected from HBE cells treated with these siRNAs. No significant differences were observed between HRV+CSE treated cells after pre-treatment with medium, transfection lipid alone or either of the two AUF-1 siRNA duplexes (Figure 5B). Although AUF-1 has been shown to associate with CXCL8 mRNA in human saliva [33], these data suggest that the 45 kDa isoform of AUF-1 is not involved in the stabilization of CXCL8 mRNA following treatment with HRV+CSE, but no firm conclusions can be drawn regarding the other isoforms. The 45 and 40 kDa isoforms of AUF-1 are purported to be involved in de-stabilizing mRNA, while the 42 and 37 kDa isoforms are involved in stabilizing mRNA [40]. Since there was a very strong basal expression of the 42 kDa isoform of AUF-1, it is unfortunate we were unable to knockdown this isoform sufficiently to determine if it was contributing to the stabilization of CXCL8 mRNA.

Transfection reagent alone or the control non-targeting siRNA (10 nM) did not have any significant effect on protein expression of KHSRP (Figure 5A). Each of the two siRNAs targeting KHSRP were significantly able to knockdown the expression of KHSRP protein, as assessed by densitometry, with a 61% and 78% knock-down compared to HRV+CSE alone (Figure 5A). CXCL8 protein level measurements revealed that KHSRP knockdown did not reverse the enhancement of HRV-induced CXCL8 by CSE (Figure 5B). One of the two siRNA duplexes had a slight, but significant, effect on enhancing CXCL8 protein levels in cells treated with HRV+CSE. Accordingly, KHSRP may be involved in dampening the expression of CXCL8, but since only one of the two siRNAs had this affect, this result is inconclusive. Although studies have shown that KHSRP associates with the 3’ UTR of CXCL8 [38,39], and is essential for rapid degradation of this transcript [39], these data suggest that KHSRP is not involved in the enhancement of HRV-induced CXCL8 by CSE. Nonetheless, it is still possible that KHSRP may be involved in globally dampening CXCL8 protein expression.

Transfection reagent alone or the control non-targeting siRNA (10 nM) also did not have any significant effect on protein expression of HuR (Figure 6A). Each of the two siRNAs targeting HuR were significantly able to knockdown the expression of HuR protein as assessed by densitometry, with a 68% and 70% knockdown compared to HRV+CSE alone (Figure 6A). CXCL8 protein level measurements revealed that HuR knock-down, using each of two different HuR-targeting siRNA duplexes, did have a significant effect on CXCL8 protein expression from HBE cells following treatment with HRV+CSE (Figure 6B). CXCL8 protein expression was reduced to levels observed with treatment of HBE cells with HRV alone (Figure 3). Compared to medium alone, HRV alone induced CXCL8 protein levels to 9.5 ± 2.4 ng/mL (Figure 3). The combination of HRV+CSE induced CXCL8 protein levels to levels above 15 ng/mL (Figures 3 and 6B). Levels of CXCL8 protein released from HBE cells following treatment with HRV+CSE in conjunction with HuR-targeting siRNA #1 or siRNA #2 were 8.9 ± 1.6 ng/mL and 9.0 ± 1.5 respectively. These data suggest that HuR is involved in stabilizing CXCL8 mRNA when HBE cells are treated with HRV+CSE, leading to elevated protein levels compared to cells treated with HRV alone.

**Figure 7:**
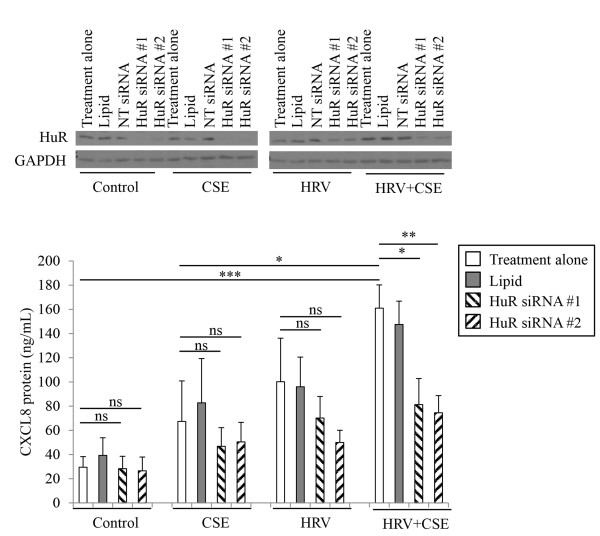
**HuR knockdown affects HRV+CSE-induced, but not basal, CSE or HRV-induced CXCL8 protein expression in HBE cells.** HBE cells were treated for 24 h prior to harvesting cell supernatants for analysis with ELISA and whole cell lysates for analysis with immunoblotting. To determine the level of HuR protein knock-down membranes were probed with a specific HuR antibody, then were subsequently stripped and re-probed with an antibody to GAPDH to ensure equal protein loading **(A**; representative of 3 separate experiments**)**. Cell supernatants were analyzed for CXCL8 protein **(B**; n=3**)**. Data are presented as mean ± SEM. Lipid denotes transfection reagent alone. NT denotes non-targeting control siRNA. Asterisks denote a significant difference between the specified treatments (*p≤0.05, **p<0.01 and ***p<0.001). ns = not significant.

In order to determine whether HuR knockdown had an effect on basal CXCL8 protein levels, or those induced by CSE or HRV treatment alone, we repeated these experiments with all four treatments. Since AUF-1 and KHSRP knockdown did not appear to play a role in HRV+CSE-induced CXCL8 we did not feel it was pertinent to extend these studies to include siRNAs targeting the knockdown of these proteins. Each of the two siRNAs targeting HuR were able to knockdown the expression of HuR protein compared to control medium, CSE, HRV and HRV+CSE alone (Figure 7A). Again, transfection reagent alone or the control non-targeting siRNA (10 nM) also did not have any significant effect on protein expression of HuR (Figure 7A). CXCL8 protein level measurements revealed that HuR knock-down, using each of two different HuR-targeting siRNA duplexes, did not have a significant effect on basal, CSE-induced or HRV-induced CXCL8 protein expression, while it did have a significant effect on CXCL8 protein expression following treatment with HRV+CSE (Figure 7B).

Collectively, these data suggests that HuR is only involved in stabilizing CXCL8 protein induced by the combined treatment of HRV+CSE but not the induction of CXCL8 by either treatment alone. This is consistent with our earlier observations that stabilization of CXCL8 mRNA decay was only observed with the combination of HRV+CSE [24]. Since CXCL8 expression can be regulated via a number of mechanisms, the induction of CXCL8 by CSE alone and HRV alone could be explained via effects at other levels than mRNA stabilization. Indeed, we have previously shown using transiently transfected CXCL8 promoter-luciferase constructs in the BEAS-2B bronchial epithelial cell line that HRV induces activation of the CXCL8 promoter [48]. It also should be noted that, since siRNA targeting HuR resulted only in partial knockdown of this protein, our data likely underrepresent the contribution of HuR to the stabilization of CXCL8 mRNA upon treatment with HRV+CSE.

In support of HuR playing a role in stabilizing CXCL8, HuR has been shown to associate with CXCL8 mRNA in human saliva [33], in monocytic THP-1 cells following stimulation with nitric oxide [34] and following an inflammatory stimulus in the BEAS-2B bronchial epithelial cell line [49]. Winzen and colleagues also show that CXCL8 mRNA is stabilized through the 3’ UTR via HuR [35]. It has also been demonstrated that both HuR and KHSRP associate with the 3’ UTR of CXCL8 but, at least in breast cancer cells following IL-1β treatment, there was a much greater association of the stabilizing factor HuR than the destabilizing factor KHSRP [38]. Since we did not see a role for KHSRP in stabilizing HRV+CSE-induced CXCL8, it is likely that HuR is also the main contributing factor in the stabilization of CXCL8 mRNA. Thus, our data add to the growing body of literature that HuR is one of the most important stabilizing factors involved in stabilizing CXCL8 mRNA in various cells types following a variety of treatments.

We recognize that our study is not without limitations. Although, CSE was used in our study rather than direct gaseous cigarette smoke exposure, CSE has been extensively used as a model for cigarette smoke exposure in tissue culture [23-26,50-52], and it is reasonable to assume that CSE mimics the soluble component of direct cigarette smoke exposure in the airway surface fluid of the lung which lines the respiratory epithelium. Relatively acute exposure to CSE may also not be completely reflective of the effects that chronic cigarette smoking has on the human airway epithelium *in vivo*, and we acknowledge that this is also a limitation of our study. In order to address some of these concerns, and to confirm our *in vitro* findings *in vivo*, we are currently performing a study using experimental HRV infections in human volunteers, comparing responses in otherwise healthy smokers and healthy non-smokers. Expression of CXCL8, as well as the mechanisms involved in its regulation will be one of the outcomes to be evaluated.

## Conclusions

We have previously reported that CSE alone and HRV alone each induce the production of CXCL8 from human bronchial epithelial cells and that when the two stimuli are combined there is at least an additive enhancement of CXCL8 compared to either treatment alone [24]. The enhancement of HRV+CSE-induced CXCL8 is regulated, at least in part, at the level of mRNA stability. Our previous studies together with our current observations provide the first demonstration that the enhanced production of CXCL8 from human airway epithelial cells exposed to the combination of HRV and CSE is regulated post-transcriptionally via mRNA stabilization and that HuR plays a key role in this process. If enhancement of CXCL8 by the combination of HRV infection and cigarette smoking is seen *in vivo*, understanding of the mechanisms behind this enhancement would aid in developing adequate treatments to limit the over-exuberant pro-inflammatory response that leads to increased neutrophil recruitment. Although not all genes regulated by HuR may be involved in the recruitment of neutrophils, it has been shown that HuR also associates with the 3’UTR of TNF-α+IFN-γ-induced neutrophil chemokines CXCL1 and CXCL2 in human airway epithelium [49]. Although the expression levels of these particular chemokines have not been investigated following treatment of airway epithelial cells with HRV+CSE, CXCL1 has been shown to be induced by HRV alone. If CXCL1 and/or CXCL2 are enhanced following HRV+CSE treatment, it is possible that HuR may be involved in this process and this offers a future avenue for investigation. Together, this study suggests that inhibition of HuR, either directly or via a pathway that increases its activation/cellular localization, may help in reducing airway epithelial production of CXCL8, limiting the excessive recruitment of neutrophils. This would be applicable not only in HRV-infected smokers but particularly in COPD patients and smoking asthmatics during HRV-induced exacerbations.

## Abbreviations

HRV: Human rhinovirus; COPD: Chronic obstructive pulmonary disease; CSE: Cigarette smoke extract; AUF-1: Adenine/uridine-rich factor-1; KHSRP: KH-type splicing regulatory protein; HuR: Human antigen R; ARE: Adenine/uridine-rich elements; TTP: Tristetraproline; BEBM: Bronchial epithelial basal medium; BEGM: Bronchial epithelial growth medium; GAPDH: Glyceraldehyde-3-phosphate-dehydrogenase; HBE: Human bronchial epithelial.

## Competing interests

The authors declare that they have no competing interests.

## Authors’ contributions

MHH designed the experiments, carried out experiments, analyzed the data and wrote the manuscript. DP conceived the study, designed the experiments, helped to analyze the data and revised and edited the manuscript. All authors read and approved the final manuscript.
